# The Child Evaluation Checklist (CHECK): A Screening Questionnaire for Detecting Daily Functional “Red Flags” of Underrecognized Neurodevelopmental Disorders among Preschool Children

**DOI:** 10.1155/2019/6891831

**Published:** 2019-12-01

**Authors:** Sara Rosenblum, Irit Ezra Zandani, Tsofia Deutsch-Castel, Sonya Meyer

**Affiliations:** ^1^Laboratory of Complex Human Activity and Participation (CHAP), Department of Occupational Therapy, Faculty of Social Welfare & Health Sciences, University of Haifa, Mount Carmel, Israel; ^2^Child Development Center, Maccabi Healthcare Services, Ashkelon, Southern District, Israel; ^3^Neurodevelopmental Service, Maccabi Healthcare Services, Haifa, Northern District, Israel

## Abstract

**Background:**

Early identification of invisible comorbid neurodevelopmental disorders, such as specific learning disorders, attention deficit hyperactive disorders, and developmental coordination disorders, is crucial to improving children's daily functional deficits related to executive functions. However, a practical questionnaire to address parents' concerns is lacking.

**Aims:**

To develop a reliable and valid assessment tool that can identify young children at risk for invisible underrecognized neurodevelopmental disorders. This article describes the development and standardization of the Child Evaluation Checklist (CHECK).

**Methods and Procedures:**

Participants were 186 children aged 3 to 6 years: 91 with suspected invisible neurodevelopmental disorders, and 95 controls with typical development. Parents completed a demographic questionnaire, the CHECK, and the Behavior Rating Inventory of Executive Function-Preschool Version (BRIEF-P).

**Outcomes and Results:**

The CHECK's construct validity indicated high internal consistency for each part (Part A: *α* = .94; Part B: *α* = .90) and moderate-to-high consistency for each of Part A's four factors. Significant correlations, as well as significant group differences, were found between the CHECK factors and BRIEF-P scores.

**Conclusions and Implications:**

Use of the CHECK allows for timely identification of suspicious (“red flags”) invisible neurodevelopmental disorders. It may support parents' sufficient awareness and knowledge to refer their children for comprehensive evaluation and intervention.

## 1. Introduction

Neurodevelopmental disorders are a group of developmental conditions characterized by developmental deficits in personal, social, academic, or occupational functioning [1]. Among these disorders are three diagnoses that are underrecognized and have high comorbidity with each other [2]. Prevalent among 3% to 20% of children [1], these diagnoses include specific learning disorders (SLD) [3, 4]), attention deficit hyperactive disorders (ADHD) [5], and developmental coordination disorders (DCD). This paper describes the development of the Child Evaluation Checklist (CHECK), a short screening tool aimed at identifying children who are at risk for these underrecognised invisible neurodevelopmental conditions. CHECK focuses on the small nuances of children's *daily functional activity performance features as related to their executive functions*. According to the Diagnostic and Statistical Manual of Mental Disorders (DSM-5) [1], SLD refers to deficiencies in reading, writing, and mathematics [6]. There is a high occurrence (40%–70%) of SLD with other developmental deficits [7], and as many as 30% to 50% of children with SLD also have attention deficits [8, 9]. DSM-5 defines ADHD as attention deficit and disruptive behavior disorders characterized by three sets of symptoms—inattention, hyperactivity, and impulsivity—with each set comprising a list of nine observable behavioral symptoms [1, 10]. Developmental coordination disorder or “clumsiness” is defined as a substantially below age-expected acquisition and execution of coordinated motor skills that significantly and persistently interferes with the activities of daily living (ADL) and affects academic/school productivity, prevocational and vocational activities, leisure, and play [11]. The DCD diagnosis is phenotypically heterogeneous, with up to 70% of children meeting criteria for at least one other neurodevelopmental disorder [12]. Despite the high commonality (5% to 10%) of DCD, and it being a significant risk factor in the long-term development of children and adolescents, it is underrecognized and underdiagnosed [11]. Although high comorbidity has been found between the abovementioned diagnoses and sensory processing deficits, this diagnosis is not yet included in DSM-5 [12–14], and therefore sensory processing deficits are not included in this study.

Regardless of the high percentage of comorbidity or cooccurrence among these three diagnoses that appear in DSM-5 (from 50% to 70%) or the complexity of their clinical manifestation [15], the general trend is to refer to each disability as a separate diagnosis.

Usually, children are diagnosed at school, around the age of 8 years or older [1, 16]. In fact, young children with SLD, ADHD, and DCD exhibit day-to-day functional deficits even before going to school, for example, in their personal hygiene, ADL, interpersonal relationships, communication, fine and gross motor activities, organization in space and time, and play and leisure [17–21]. Such deficits may concern both children and adults and negatively impact the child and their whole family [18, 22, 23]. Previous literature indicated that people with invisible neurodevelopmental disabilities feel already from very early that “Something with me or my child is not the same as others” in reference to their daily functional capabilities. Nevertheless, a tool that can combine all the pieces of daily functional evidence to one whole picture of functional deficits tied to invisible neurodevelopmental disabilities is lacking [17–23]. Theoretical models and research findings have previously connected these daily functional deficits with deficient executive functions (e.g., [24, 25]). The term “executive function” refers to a neuropsychological process that enables physical, cognitive, and emotional self-control [26]. The hierarchical hybrid model of executive functions developed by Barkley [27] places inhibition at the top of the hierarchy. Other executive functions including nonverbal working memory; internalization of speech (verbal working memory); self-regulation of affect, motivation, and arousal; and reconstitution are placed at the lower level [27]. Together, these skills impact the functional cognition performance used to accomplish essential activities in daily life [27, 28]. Efficient executive functions provide children efficient day-to-day functioning while managing age-related daily tasks, coping with challenges, and solving problems [29]. Executive functions are also described as body functions in the International Classification of Functioning, Disability, and Health (ICF) [30], a conceptual and operational framework published by the World Health Organization. The ICF describes multidirectional interactions between body functions and structures, activities, and participation, while considering both environmental and personal factors. Deficient executive functions have been described among children and adults with SLD [31], ADHD [32], and DCD [33]. Furthermore, executive function-related deficiencies were described in these populations related to their body and motor control [34], verbal abilities [35], self-regulation and control, and social abilities [36–38]. Such deficiencies may appear very early in life in the way children engage in daily functional activities. Furthermore, they predict functional impairments later in life, limiting participation in various life areas [39–41].

Children suspected with neurodevelopmental invisible disabilities are at significant developmental risk in the long term. Their performance abilities, self-esteem, and wellbeing may be negatively affected [25, 42]. Despite knowing the importance of the early detection of executive function deficiency in daily functioning among younger children, most daily functional delays among children suspected for neurodevelopmental invisible disabilities are diagnosed (if at all) only when the child reaches school age. Thus, this phenomenon remains underrecognized [1, 16]. Knowledge is scarce about how deficient executive functions can impact these children's performance in daily activities, especially in preschool ages. Furthermore, it is important to consider gender differences. Especially at young ages, boys are generally more extroverted than girls and, thus, may reflect signs of impaired daily function abilities in their behavior, whereas girls are more introverted and may express their frustration less often and more verbally [43].

Early identification is therefore important to prevent future emotional problems, such as reduced self-esteem, anxiety, and depression, as well as social and behavioral problems [44, 45]. There are several screening questionnaires for detecting developmental delay among children aged from birth to five that focus on physical, cognitive, linguistic, and social-emotional growth and development [46]. One parent questionnaire is the Behavior Rating Inventory of Executive Function: Preschool Version (BRIEF-P) that assesses executive functions [47]. The Child Evaluation Checklist (CHECK) adds to those existing tools by providing the option of identifying children ages 3 to 6 years at risk for invisible neurodevelopmental disorders through a short, easy to complete parent report about their children's day-to-day functioning, with specific emphasis on executive functions. Both the complexity and latency were considered for enabling identification, even before a physician provides a specific diagnosis according to the DSM-5 criteria [48].

As such, this current study's research hypotheses are as follows: (1) The CHECK's construct validity will be established by the factor analysis for Parts A and B, and each factor will show adequate internal reliability (*α* > .70). (2) Significant correlations will be found between the CHECK scores and all five BRIEF-P subscale scores (inhibition, shift, emotional control, working memory, and planning), thus establishing concurrent validity. (3) Significant differences will be found in the CHECK scores, beyond gender and age, between children diagnosed by a pediatrician as suspected for invisible neurodevelopmental disorders and those with typical development.

## 2. Materials and Methods

### 2.1. Questionnaire Development and Content Validity Determination

The CHECK is a one-page questionnaire designed for use by parents to provide information about their children's ability to function within the context of their natural environments during the previous three months. The questionnaire was developed based on a number of resources that established the tool's content validity: (a) the DSM-5 definitions of SLD, ADHD, and DCD [1], (b) the ICF framework, which served as the basis for understanding interactions between components that reflect functioning [30], (c) Barkley's hybrid model of executive functions [27], (d) the current literature about daily functional challenges of children with these conditions, (e) previously designed screening questionnaires (e.g., [49, 50]), and (f) analysis of interviews with parents of children and adults with SLD, ADHD, and DCD about their children's daily functioning experiences, confrontations, and challenges (e.g., [17, 51–54]). Initially, based on these resources and the researchers' extensive clinical experience, including observations on young children, 48 statements were formulated by the first author to reflect daily routine functions that challenge children aged 3 to 6 years with suspected invisible disabilities because of the need for executive function involvement in their performance (i.e., ADL, communication, inhibition and self-regulation, and organization in space and time). Each statement was associated with at least one of the main concepts of the ICF [30]. For example, “Can solve problems created in play\wardrobe” classified as body functions, and “Does his\her needs independently, in comparison with what's expected of children of his\her age” classified as activities and participation. The 48-statement questionnaire was sent to three physicians and five senior occupational therapists, all experts in child development. To establish content validity, the experts were asked to comment whether (1) each item is appropriate for detecting functional deficiency related to executive functions among children with suspected invisible neurodevelopmental disabilities or whether (2) the items were clearly worded. There was 100% expert agreement for 40 items but only 60% agreement for eight items that were consequently deleted. Additionally, the wording of five items was improved following expert recommendations. For example, in Item 15, “Organizes body for activity,” the following examples were added in parenthesis for clarity (i.e., jumping, skipping, and throwing a ball).

The 40-item questionnaire was then sent to two other expert pediatric occupational therapists with 20 years clinical experience, and three occupational therapy researchers experienced in populations with invisible disabilities across the lifespan. A second round of content expert validity was performed based on their feedback. Following the expert input, the wording of three items was again improved and 100% agreement was achieved for the final 40 items.

The CHECK's final version is divided into two parts. Part A includes 30 items that entail the domains previously described that are related to various daily activities (e.g., Item 7, “Eats in a manner suiting his\her counterparts, such as cleanliness, tidiness, control over utensils” or Item 2, “Understands instructions he\she is provided”). Parents are asked to score the frequency in which the item describes their child: always (4), often (3), rarely (2), or never (1). Part B includes 10 sentences about the child's global performance level related to various executive function outcomes reflected in daily function. Items are rated on a scale from 1 (very low performance level) to 5 (high performance level) compared with that of the child's typically developing peers. A higher score represents better performance. Examples from Part B include Item 2, “In comparison with other children, the child's attention and concentration ability is …” or Item 6, “The child's adaptation ability to changes in routine is …”

### 2.2. Participants

A required sample size of 135 participants was calculated with the G∗Power statistical program, based on a moderate effect size (*f*^2^(*V*) = .0625, alpha = .05, Power = .80). Children previously diagnosed with an intellectual, physical, or neurological disability were excluded. Initially, 96 children diagnosed with suspected invisible neurodevelopmental disorders participated in the study. Those children were referred by their family physician or pediatrician to a child developmental center because their parents or teachers were concerned that the children were not performing like other children. A developmental pediatrician confirmed the parents' concerns and defined the children as suspected for invisible neurodevelopmental disorders (SLD, ADHD, and DCD symptoms) based on the DSM-5 criteria [1]. This developmental pediatrician did not determine a specific diagnosis but recommended follow-up with or intervention by a pediatrician or occupational therapist. Another group of 95 children with typical development were recruited from the same kindergartens or communities as the children with invisible disabilities through a chain-referral sampling method. Parents completed a demographic questionnaire and reported neither difficulties in daily functioning nor the need to be referred to a health or educational professional because of any developmental functional concerns. Participants were then matched for age, gender, and socioeconomic level as reflected in the mothers' years of education (ranged from 9 to 20 years). Previous research has found that the level of mother's education and socioeconomic status can impact their child's development because of the learning opportunities and possibilities that are directed by the mother-child interaction [55].

The results presented hereafter refer to 186 children (91 children with suspected invisible neurodevelopmental disorders and 95 with typical development) aged 3 to 6 years—141 (75.8%) boys and 45 (24%) girls.

### 2.3. Instruments

#### 2.3.1. BRIEF-P

The BRIEF-P [47] consists of 63 items related to behavioral manifestations of executive functions, rated on a 3-point scale indicating whether the behavior occurs never (1), sometimes (2), or often (3). The items are divided into five scales of executive functions (inhibitory control, shifting, emotional control, working memory, and planning and organization) that produce three index scores (inhibitory self-control, flexibility, and emergent metacognition). The sum of the clinical scales reveals the global executive composite. In addition, two validity scales were obtained. The inconsistency scale aims to determine if the respondent has answered in an especially conflicting manner, and the negativity scale measures whether the respondent answered in an unusually pessimistic manner. Higher scores indicate more dysregulation in behaviors associated with executive functions.

### 2.4. Procedure

The Health Care Service Human Research Ethics Committee (Helsinki approval No. 2009087), as well as the Israeli Ministry of Education (No. 506/7902) and the Institutional Ethics Committee (No. 320/13), authorized the study. All parents signed informed consent forms and were then asked to complete a demographic questionnaire, the BRIEF-P, and the CHECK.

### 2.5. Data Analysis

Data were analyzed using SPSS version 22 and descriptive statistics to describe the participants. To verify the CHECK's construction and dimensions based on the theoretical and clinical experience of the CHECK's developer, exploratory factor analysis was conducted using principle components to find the factors of Parts A and B. The number of extracted factors in each part was chosen based on a screen plot of the eigenvalues and on factor interpretability.

The resulting factor solution was subsequently rotated by means of an oblique (Oblimin) rotation procedure due to the possible correlation of the factors which all represent functional reflections of executive function deficiency. Item-factor loading with values of at least .35 were deemed salient. All items that did not meet this criterion were dropped, as were all items that loaded highly on multiple factors. Internal consistency reliability was evaluated using Cronbach's alpha coefficient.

After confirming the CHECK's final format, Pearson's correlation analyses were performed on the entire sample to better understand the relationship between the CHECK factors and BRIEF-P subscale scores and to establish concurrent validity. Consequently, gender differences and differences between children with invisible disabilities and those with typical development were analyzed across the CHECK factors via MANCOVA analysis, holding age as the covariate. Univariate ANCOVA analyses were used to determine the source of the group differences. The ANCOVA was performed to check for group differentiation of the final CHECK scores while holding age as covariate.

## 3. Results and Discussion

### 3.1. Examination of the Questionnaire Validity and Reliability

#### 3.1.1. Construct Validity 1: Exploratory Factor Analysis for CHECK Part (A and B)

*(1) CHECK Part A: Child's Daily Function Nuances as Reflectors of Executive Function Abilities*. Analysis of Part A revealed four distinct factors, comprised of 30 items, with eigenvalues greater than 1 (Table 1). The four factors yielded a cumulative variance percentage of 54.05%, with an internal consistency of *α* = .94.

The four factors, as well as the internal consistency reliability measured by the coefficient alpha of each factor are as follows:

1. The first factor, *expression/performance management*, included 12 items and accounted for 37.23% of the variance with *α* = .91
2. The second factor, *self-regulation*, included six items and accounted for 6.31% of the variance with *α* = .79
3. The third factor, *organization—body, essentials, social*, included nine items and accounted for 5.89% of the variance with *α* = .88
4. The fourth factor, *ADL*, included three items and accounted for 4.67% of the variance with *α* = .68

*(2) CHECK Part B: Child's General Daily Function Compared to Others*. The analysis revealed one distinct factor with eigenvalues > 1, comprised of 10 items (Table 2). The cumulative percentage of this one factor was 59.33% with an internal consistency of *α* = .92.

#### 3.1.2. Internal Consistency Reliability

As presented in Tables 1 and 2, Cronbach's alpha coefficient—calculated separately for each part—indicated excellent internal consistency for each (Part A, 30 items: *α* = .94; Part B, 10 items: *α* = .92). Sufficient-to-high levels of internal consistency were achieved for each factor in Part A, ranging from *α* = .68 to *α* = .91.

#### 3.1.3. Concurrent Validity of the CHECK with the BRIEF-P: Entire Sample (*N* = 186)

As shown in Table 3, significant negative low-to-moderate correlations (*r* = −.23 to *r* = −.62, *p* < .001) were found between the four factors of Part A (cognitive-language, self-regulation, organization, and ADL), the CHECK Parts A and B final scores with the three BRIEP-P indexes (inhibitory self-control, flexibility, and emergent metacognition), and the global executive composite and each subscale (inhibition, shift, control, memory, and planning).

#### 3.1.4. Construct Validity 2

In this phase, construct validity of the CHECK factors was examined by analyzing gender differences and group differences (children with suspected invisible neurodevelopmental disorders and controls). Although no significant difference was found in age between groups, age was held as the covariate because the significance level was borderline (*p* = .062). Pearson's correlations were conducted to test the correlation between age and CHECK factors showing a weak positive correlation (*r* = −.25 to *r* = −.30, *p* < .001).

As presented in Table 4, initial analysis indicated no significant group differences in participants' sociodemographic variables, such as children's age and gender or mother's education.

The MANCOVA analysis indicated significance differences across age (*F*_(4,178)_ = 8.63, *p* < .001, partial *η*^2^ = .162), no significance across gender (*F*_(4,178)_ = 1.88, *p* = .12, partial *η*^2^ = .040), and significant group differences (*F*_(4,178)_ = .11.40, *p* < .001, partial *η*^2^ = .204) in Part A. Furthermore, no significant gender or group interaction was found (*F*_(4,178)_ = 1.37, *p* = .246, partial *η*^2^ = .03). The subsequent ANCOVA analysis for the four CHECK factors indicated significant age contribution for language and cognition (*F*_(1,181)_ = 19.05, *p* < .001, partial *η*^2^ = .10), organization (*F*_(1,181)_ = 15.48, *p* < .001, partial *η*^2^ = .08), and ADL (*F*_(1,181)_ = 19.01, *p* < .001, partial *η*^2^ = .10). No significant contribution was found for emotional regulation (*F*_(1,181)_ = 1.43, *p* = .234, partial *η*^2^ = .01). Results of the ANCOVA analysis for the four CHECK factors across gender and groups are presented in Table 5. In addition, ANCOVA analysis for the final scores of CHECK Parts A and B showed a significant effect of age to the final CHECK A and B scores (*F*_(1,181)_ = 20.81, *p* < .001, partial *η*^2^ = .10; *F*_(1,181)_ = 21.54, *p* < .001, partial *η*^2^ = .10, respectively).

## 4. Discussion

This study describes the development of CHECK—a quick, easy-to-use, and practical screening tool focused on children's daily functional characteristics. As such, it is suitable for use with parents. CHECK responds to the need for assessment tools that address the concepts of *activities* and *participation*, defined by the International Classification of Functioning Disability and Health [30] as central concepts related to each individual's health.

The four factors achieved for Part A of CHECK support the study's results presented in the Introduction and reflect the children's daily challenges. The items in the first factor reflect how the children express themselves and how they manage their daily performance. Domains required at this phase are attention (Item 1), understanding instructions (Item 2), memory (Item 3, remembering stories), and correctly estimating the difficulty of a task (Item 25). The children then perform the daily tasks while managing their performance, thus self-verbalizing (Item 4), talking about school (Item 9), expressing thoughts (Item 10), and doing the activities in a reasonable time (Item 11), but also correcting themselves when wrong (Item 25), solving problems if they occur (Item 8), taking responsibility (Item 18), and completing the task (Item 19). Such a process of expression, performance, and monitoring is based on a combination of executive function abilities such as attention, inhibition, initiation, working memory, shifting, and planning and organization [40]. Among the items in this factor are verbal skills that allow children to learn, understand, and interpret their physical, social, and conceptual worlds [56]. Indeed, verbal delay may be one of the first reasons for parents' concern about their children's development and for seeking professional help [56, 57]. After preparation and performance management, the second factor reflects the self-regulation and control abilities required for success in daily demands. The literature has described varied self-regulation and adaptive functioning aspects, such as cognitive skills, executive functions, emotions, and motivation [58]. Thus, the second factor includes items that reflect the children's ability to self-regulate, not be impulsive, be generally calm, calm themselves, cope with changes, and get out of bed willingly.

At preschool age, children's ability to regulate their behavior relates to school readiness [59, 60]. Because deficits in self-regulation, self-control, and self-monitoring were described among children and adults with ADHD [61–63] and SLD [64], identifying the deficiency early in the child's development may lead to appropriate strategies to prevent future failure.

The third factor uniquely combines body organization (including pencil control) and social behavior under the umbrella term of *organizational abilities*. This ability, described by Godefroy [65] as an executive function domain, is the ability to organize thoughts to efficiently plan and carry out activities in the correct sequence and tempo within the given time range and space [66, 67]. Thus, organizational abilities are required to efficiently execute body motion (Items 15, 20, and 21; [33]), converse a message (Item 12), and play and communicate with friends (Items 13, 14, and 17), as well as control a pen or small object (Items 22–23). For example, controlling small objects requires gentle organization and motor adjustments of the small intrinsic hand muscles in space and time, defined as *in-hand manipulation* [68], for efficient skilled performance. Because organizational abilities such as dysgraphia are significantly inferior among children with SLD, ADHD, and DCD and significantly correlated with their handwriting proficiency [69], more emphasis needs to be given to this skill in the preschool years. Thus, deficient organization abilities may be reflected in how children organize their bodies and control objects in their play and social behavior. In all, these three CHECK factors successfully represent the action control necessary for task execution [58].

The final CHECK factor consists of ADL items, which are the basic tasks children learn to perform by themselves as they develop and become more independent. Performing these skills provides children with self-competence and is important for both the children and the family atmosphere, especially in the morning when getting organized to leave home for school and work [70].

The medium-to-high internal reliability values found for these factors (range *α* = .68‐.91), as well as for the entire Part A (*α* = .94), indicates that these items successfully reflect daily functional challenges among children aged 3 to 6 years with suspected invisible neurodevelopmental disorders. Furthermore, it indicates that parents are able to report successfully those items as reflecting their children's functional abilities. As shown in the high internal reliability achieved for Part B (*α* = .92), parents are also able to rank their children's function well as compared to other children based on the 10 items the authors chose to include in this part. Those results support previous findings about the accuracy of the information parents supply when asked appropriate questions to identify “red flags” in their children's daily functional abilities [71, 72]. However, previous studies have indicated that 3 to 6 years pass between the time when parents sense that something about their child is not the same as other children and the time a diagnosis is given [73]. Thus, it is important to provide opportunities to identify invisible-disability developmental delays in the preschool years.

During the preidentification period, negative influences may appear in the children's and the parent's functional and emotional experiences, as well as in the interactions between parents and their children [51]. Children's frustration and sense of failure, accompanied by family strains, can cause secondary socioemotional, social, and family problems and deficient participation and self-perception [74–77]. Hence, obtaining knowledge from parents by means of a standardized early screening tool based on the children's abilities, as reflected in daily activity performance, can be valuable towards this essential identification of “red flags.”

Further evidence for the benefits of CHECK in detecting functional deficits related to executive functions was achieved through the concurrent validity results. Significant negative low-to-medium correlations (*r* = −.27 to −.57) were found between CHECK's three action control factors and executive function domains (i.e., inhibitory control, shifting, emotional control, working memory, and planning and organization). Specifically, the high correlation level between both CHECK parts and the BRIEF-P working memory and planning abilities (*r* = −.50 to −.67) align with previous literature stating that deficits in these executive functions may cause deficient daily functioning among children [70]. Interestingly, the lowest significant correlation was found with ADL performance (i.e., independent lavatory, independent dressing, and clean eating; *r* = .16‐.26). Indeed, ADL performance at a young age requires learning the order and sequence of the activities to be performed, and there is a need to implement executive functions while using the lavatory, dressing, or eating. However, those specific activities all have less complex demands than other life tasks (such as those described in the other three factors).

The significant group differences found for all CHECK factors and the final scores while considering age indicate the sensitivity of the scale in distinguishing between children at risk for invisible neurodevelopmental disorders and those not at risk. In fact, deficient abilities in specific tasks were reported in this population concerning how they manage their actual performance [77–79], self-regulation and self-control [56], and organization abilities [2, 33, 68]. Furthermore, significant relationships were found between children's executive function abilities and their instrumental ADL [77], play [21], and physical activity performance [80].

The results of this study also indicate the need to consider gender differences when attempting to identify children suspected for neurodevelopmental invisible disorders. Significant differences were found between boys and girls in their organization abilities for their bodies and objects, as well as social organization, in favor of girls. On one hand, this suggests that boys have more deficiencies in this area; on the other hand, it challenges identification of girls who, although they tend to be more organized, may confront invisible disabilities.

More studies are needed to further establish the CHECK reliability and validity; however, these primary results enable its practical clinical use as a screening tool. Considering the emotional and social consequences of “invisible disabilities” for children, parents, and families [81], early identification is crucial. Parents are in a position to observe their children over time and across settings and are the best source of information about their children [80]. They are often the first to recognize something concerning about their child's development and therefore seek further professional services from pediatricians. However, although screening questionnaires can provide important information, they serve only as a basis for a more comprehensive evaluation by proficient clinicians [82]. Nevertheless, this study's findings provide encouraging indications that CHECK may be a useful screening tool to identify “red flags” for children aged 3 to 6 years with neurodevelopmental invisible disability characteristics through parents' reports about their children's daily functioning.

Using CHECK can provide parents and the education team with a tool to see each child and his or her needs. It not only highlights differences in the child's daily performance compared to other children, but also more specifically defines the nature of the difficulty. Furthermore, because of the complexity and comorbidity of invisible neurodevelopmental disorders, CHECK may aid in observing individual differences, strengths, and weakness throughout development, while identifying not only disabilities but also *abilities* [15, 81, 83].

## 5. Conclusions

The current study confirms that the CHECK scale, which is efficient, short, and easy to use, can reveal reliable and valid information concerning the presence of subtle early signs of possible future SLD, ADHD, or DCD at the ages of 3 to 6 years. Therefore, the use of CHECK can increase early detection of such neurodevelopmental disorders among children and help professionals refer children for comprehensive evaluation and further intervention with caution, while considering normal variation [52].

## 6. Limitations and Future Research

The children in this study were all from the northern region of the country and were referred to a regional child developmental center. The control group of children with typical development were recruited by a chain-referral sampling method. Although parents reported no developmental concerns, these children did not undergo a developmental assessment. Given that Part B items achieved the highest loading values, it is questionable whether asking worried parents specifically about their child's *general function*, *attention capacity*, and *work habits* in comparison to those of peers will lead to more a comprehensive evaluation. Although CHECK exhibited a good level of internal construct and concurrent validity, it is important to emphasize that the clinical validity of any measure requires testing over time and implementation across a variety of larger sample groups. In addition, a longitudinal study is required to discover whether the children suspected for invisible neurodevelopmental disabilities (as identified by CHECK) were indeed diagnosed with ADHD, SLD, or DCD later in childhood.

## Figures and Tables

**Table 1:** CHECK factor loading of questionnaire items and internal consistency: Part A.

Item	Description	Factor			
		Expression and performance management	Self-regulation	Organization	ADL^a^
10	Expresses thoughts	.777			
3	Remembers stories	.772			
4	Verbalizes him/herself	.758			
2	Understands instructions	.715			
9	Talks about school	.699			
18	Takes responsibility	.645			
25	Corrects him/herself when wrong	.564			
1	Attentive	.520			
30	Correctly estimates difficulty of task	.520			
8	Solves problems in play/dressing	.514			
19	Completes tasks	.473			
11	Does activities in reasonable time	.359			
27	Is generally calm		.683		
29	Calms him/herself down		.641		
26	Gets out of bed willingly		.618		
16	Deals with changes		.497		
24	Is not impulsive		.445		
28	Sleeps well at night		.355		
15	Organizes body for activity			.842	
21	Has good balance			.828	
20	Has good coordination			.727	
22	Has good in-hand control of small objects			.627	
23	Has good pencil control; writes and draws what he/she wants			.551	
13	Cooperates with friends			.524	
14	Gets ready for a game			.505	
12	Communicates properly to get what he or she wants			.486	
17	Is likable among friends			.435	
6	Is independent in the lavatory				.762
5	Is independent dressing				.732
7	Eats cleanly and in order				.703
	Eigenvalue	11.16	1.89	1.77	1.38
	% of variance	37.23	6.31	5.89	4.89
	Internal consistency (*α*): entire sample (*N* = 186)	.91	.79	.88	.68
	*α*-Children with suspected disorders (*n* = 91)	.91	.67	.81	.73
	*α*-Children with typical development (*n* = 95)	.73	.68	.83	.69

*Note*. *N* = 186. ^a^Activities of daily living.

**Table 2:** CHECK factor loading of questionnaire items and internal consistency: Part B.

Item (compared to other children):	Loading
General functioning	.854
Attentional capacity	.850
Work habits	.819
Initiation capability	.794
Inhibition	.792
Emotional domain	.774
Verbal communication	.752
Social domain	.751
Memory capacity	.691
Adapt to changes	.588
Eigenvalue	5.930
% of variance	59.330
Internal consistency (*α*): entire sample (*N* = 186)	.920
*α*-Children suspected disorders (*n* = 91)	.91 0
*α*-Children with typical development (*n* = 95)	.76 0

*Note*. *N* = 186.

**Table 3:** Correlations between four CHECK factors: Parts A and B final scores, entire sample.

	CHECK					
	Factor				Part total	
BRIEF	Expression and performance management	Self-regulation	Organization	ADL	A	B
Inhibitory control	-.427^∗∗^	-.624^∗∗^	-.431^∗∗^	-.241^∗∗^	-.529^∗∗^	-.419^∗∗^
Shifting	-.276^∗∗^	-.408^∗∗^	-.345^∗∗^	-.160^∗^	-.367^∗∗^	-.349^∗∗^
Emotional control	-.360^∗∗^	-.495^∗∗^	-.397^∗∗^	-.253^∗∗^	-.457^∗∗^	-.356^∗∗^
Working memory	-.669^∗∗^	-.497^∗∗^	-.574^∗∗^	-.252^∗∗^	-.668^∗∗^	-.587^∗∗^
Planning and organization	-.548^∗∗^	-.425^∗∗^	-.540^∗∗^	-.252^∗∗^	-.583^∗∗^	-.502^∗∗^
ISCI^a^	-.433^∗∗^	-.621^∗∗^	-.453^∗∗^	-.265^∗∗^	-.542^∗∗^	-.425^∗∗^
FI^b^	-.656^∗∗^	-.493^∗∗^	-.590^∗∗^	-.264^∗∗^	-.669^∗∗^	-.584^∗∗^
EMI^c^	-.356^∗∗^	-.510^∗∗^	-.419^∗∗^	-.229^∗∗^	-.464^∗∗^	-.393^∗∗^

*Note*. *N* = 186. ^a^Inhibitory self-control index; ^b^flexibility index; ^c^emergent metacognition index. ^∗^Correlation is significant at the 0.05 level (2-tailed). ^∗∗^Correlation is significant at the 0.01 level (2-tailed).

**Table 4:** Comparison of demographic characteristics of children suspected for neurodevelopmental disabilities and those with typical development.

Variable	Children with suspected neurodevelopmental disabilities (*n* = 91)	Children with typical development (*n* = 95)	*t*	*p*
	*M* (SD)	*M* (SD)		
Child's age (months)	53.88 (9.27)	54.06 (9.24)	.89	.062
Mother's education (years)	14.35 (2.41)	14.94 (2.07)	1.79	.886
			*χ* ^2^	*p*
Gender				
Boys	72 (79.1%)	69 (72.6%)	1.07	.301
Girls	19 (20.9%)	26 (27.4%)		

**Table 5:** Gender and group differences across four CHECK factors and Parts A and B final scores.

Factor	Gender	Control group (*n* = 95)	Suspected invisible disabilities group (*n* = 91)	Total gender	Gender *F*_(1,181)_ (*η*_*p*_^2^)	Group *F*_(1,181)_ (*η*_*p*_^2^)	Gender∗group *F*_(1,181)_ (*η*_*p*_^2^)
Expression and performance management	Boys^a^ (*n* = 141)	3.54 (.31)	3.04 (.52)	3.29 (.49)	2.49	34.45^∗∗∗^	1.97
	Girls^b^ (*n* = 45)	3.58 (.27)	3.26 (.52)	3.45 (.42)	.01	.016	.01
	Total^c^ (*N* = 186)	3.55 (.30)	3.09 (.52)				

Self-regulation	Boys^a^	3.47 (.39)	3.16 (.44)	3.31 (.45)	2.37	7.88^∗∗^	2.20
	Girls^b^	3.49 (.45)	3.39 (.46)	3.44 (.45)	.01	.04	.01
	Total^c^	3.48 (.40)	3.21 (.46)				

Organization	Boys^a^	3.73 (.31)	3.18 (.50)	3.45 (.50)	7.15^∗∗^	36.60^∗∗∗^	5.17^∗^
	Girls^b^	3.79 (.26)	3.52 (.45)	3.67 (.37)	.04	.17	.03
	Total^c^	3.75 (.29)	3.25 (.51)				

ADL^d^	Boys^a^	3.66 (.34)	3.34 (.62)	3.50 (.53)	0.12	7.80^∗∗^	0.99
	Girls^b^	3.64 (.51)	3.47 (.60)	3.57 (.55)	.00	.04	.01
	Total^c^	3.65 (.39)	3.37 (.61)				

CHECK A final score	Boys^a^	3.60 (.27)	3.13 (.40)	3.36 (.41)	4.86^∗^	39.05^∗∗∗^	4.24^∗^
	Girls^b^	3.63 (.24)	3.39 (.42)	3.53 (.35)	.03	.18	.02
	Total^c^	3.61 (.26)	3.19 (.42)				

CHECK B final score	Boys^a^	4.17 (.62)	3.41 (.71)	3.78 (.77)	0.89	38.40^∗∗∗^	0.49
	Girls^b^	4.25 (.53)	3.62 (.84)	3.98 (.74)	.01	.17	.00
	Total^c^	4.19 (.60)	3.46 (.74)				

*Note*. ^a^*n* = 141; ^b^*n* = 45; ^c^*N* = 186. ^d^ADL = activities of daily living.

## Data Availability

The data used to support the findings of this study have not been made available because they are restricted by the The Health Care Service Human Research Ethics Committee (Helsinki approval No. 2009087), as well as the Israeli Ministry of Education (No. 506/7902) in order to protect patient privacy. Data are available from Dr. Tsofia Deutsch-Castel, deutsh_t@mac.org.il for researchers who meet the criteria for access to confidential data.
